# Young Male Presenting with an Acute Diarrheal Illness with Unexplained Transudative Ascites: An Atypical Presentation of Appendicular Tuberculosis

**DOI:** 10.1155/2020/8835081

**Published:** 2020-07-16

**Authors:** Chamara Dalugama, Achila Jayasinghe, Shamali Abeygunawardena, Manoji Pathirage, Thilak Jayalath, Udaya Ralapanawa, S. S. Jayasundara, Sulochana Wijetunga

**Affiliations:** ^1^Department of Medicine, University of Peradeniya, Peradeniya, Sri Lanka; ^2^Teaching Hospital, Peradeniya, Sri Lanka; ^3^Department of Pathology, University of Peradeniya, Peradeniya, Sri Lanka

## Abstract

**Introduction:**

Appendicular tuberculosis is a rare form of extrapulmonary tuberculosis involving the gastrointestinal tract. Diagnosis of appendicular tuberculosis is difficult due to its atypical presentation. Histological confirmation remains the gold standard in diagnosis. *Case Presentation*. We report a 37-year-old Sri Lankan male presenting with a diarrheal illness with high fever for 8 days in the background of constitutional symptoms for 1-month duration. He was pale and had moderate amount of free fluid in the abdomen. Inflammatory markers were elevated, and CT abdomen revealed a thickened elongated appendix. Diagnostic paracentesis revealed a lymphocytic transudative ascites. A macroscopically minimally inflammed appendix removed at laparotomy and histology confirmed presence of tuberculous granulomata with caseation. He made an uneventful recovery by the anti-tuberculous therapy.

**Conclusion:**

High degree of suspicion is needed in diagnosis of appendicular tuberculosis due to its nonspecific presentation, and we emphasize the need of histological assessment of the appendix resected for the case of clinical appendicitis, as it may prompt the diagnosis of a rare but treatable case of tuberculosis.

## 1. Introduction

Extrapulmonary tuberculosis still remains a greater diagnostic challenge to the treating physicians despite advancement is medical diagnostics. Intestinal tuberculosis is a form of extrapulmonary tuberculosis with very nonspecific presentation leading to delayed diagnosis. Tuberculosis involving the appendix can occur in association with adjacent ileocecal region but very rarely in isolation. An unexplained lymphocytic exudative ascites would prompt a physician to consider tuberculosis, but a transudative ascites might make it less likely. We report a rare case of isolated appendicular involvement of tuberculosis in a young male with transudative ascites.

## 2. Case Presentation

A 37-year-old previously healthy Sri Lankan male pharmacist presented to a tertiary care hospital with 8-day history of intermittent high spiking fevers. He has developed frequent small amounts of watery loose stools for the similar duration but denied blood and mucus in stools or associated abdominal pain or tenesmus. He did not have cough, hemoptysis, dysuria, or headache in systemic inquiry. He described feeling unwell for a last one-month period with significant loss of appetite and weight loss of 6 kg over the 1-month period. He did not have any chronic medical illness such as diabetes mellitus, hypertension, or dyslipidaemia. He denied a past history or a contact history of tuberculosis of a family member although he was exposed to many people as a pharmacist while dispensing medications.

On examination, we found an averagely built male who is moderately pale, but not icteric. He did not have a clinically significant lymph node or thyroid enlargement. His pulse rate was 120/minutes with a blood pressure. He was tachycardic with a pulse rate of 120/minute and a blood pressure of 130/80 mmHg. His precordial examination revealed normal heart sounds with no murmurs. He was tachypnic, but lung examination was unremarkable. The abdomen was distended with mild tenderness in the right lower quadrant without hepatosplenomegaly. There was moderate amount of free fluid in the abdomen.

His initial blood workup revealed a hemoglobin level of 9.5 g/dL, white count of 16 ∗ 10^6^/L (neutrophils 70%), and a platelet count of 560 ∗ 10^6^/L. His erythrocyte sedimentation rate is 120 mm in 1^st^ hour, and C-reactive protein level is 290 mg/L. Alanine transaminase (ALT) level was 112 U/L, and aspartate transaminase (AST) level was 88 u/L. Alkaline phosphate level was 230 u/L. Serum bilirubin level was normal. Urine analysis was normal. Three blood cultures and a urine culture did not isolate any pathogen. Chest radiograph was normal. Two-dimensional echocardiogram revealed no murmurs or pericardial effusion.

**Figure 1:**
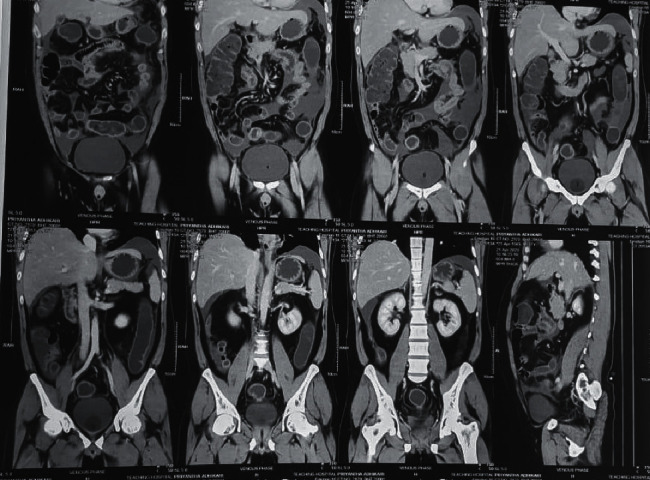
Coronal reconstructed images of the abdomen and pelvis.

**Figure 2:**
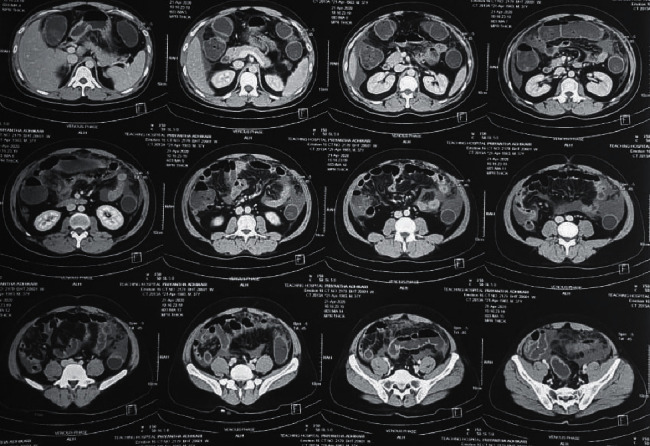
Transverse sections through the abdomen and pelvis demonstrating long thickened retrocecal appendix with minimal inflammation and moderate amount of free fluids.

Peritoneal fluid analysis revealed 15 white cells/*μ*L (80% lymphocytes), protein level of 2 g/dL, and LDH level of 190 IU/L. Peritoneal fluid was negative for acid fast bacilli. TB PCR of the peritoneal fluid was negative. The serum-ascites albumin gradient (SAAG) was 1.5 g/dL (serum albumin: 3.5 g/dL). CECT abdomen revealed a long thickened retrocecal appendix with minimal inflammation and moderate amount of free fluids (Figures 1 and 2). An explorative laparotomy was performed subsequently, and a mildly inflammed retrocecal appendix was found and removed.

**Figure 3:**
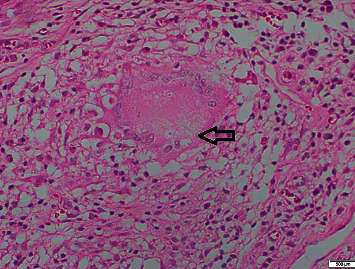
High-power view (×400) of the serosa and the mesoappendix shows numerous granulomata composed of epithelioid cells, Langerhans giant cells, and lymphocytes with central spotty caseous necrosis. H&E stain. The black arrow points at caseating granuloma.

**Figure 4:**
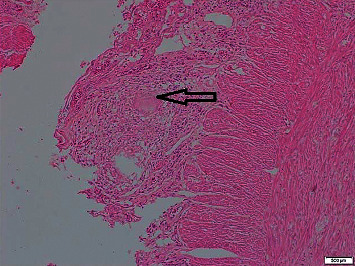
Midpower view (×100) of the serosa and the mesoappendix shows numerous granulomata composed of epithelioid cells, Langerhans giant cells, and lymphocytes with central spotty caseous necrosis. H&E stain. The black arrow points at caseating granuloma.

Microscopically acute inflammation was not present. Serosa and mesoappendix show numerous granulomata composed of epithelioid cells and Langerhans giant cells. Many show central spotty caseous necrosis (Figures 3 and 4).

Initially, the patient was treated with merapenum, ofloxacin, and metronidazole for presumed gastrointestinal sepsis for 8 days until histology was available. Although he had a mild clinical response to antibiotics, he continued to spike fevers and inflammatory markers remained elevated. The patient was started on anti-tuberculous treatment (ATT) and a short course of oral dexamethasone. His anti-tuberculous treatment regime included isoniazid, rifampicin, pyrazinamide, and ethambutol in the intensive phase, and isoniazid and rifampicin combination to continue in the continuation phase.

He made an uneventful recovery with marked clinical improvement following commencing of ATT. In two weeks of follow-up visit, he was well with a weight gain of 3 kg and inflammatory markers were normalized.

## 3. Discussion

Tuberculosis is an ancient infection dating back to thousands of years affecting humankind, worldwide around 10 million people will become symptomatic with tuberculosis each year, and one-fourth of the world population is infected with tuberculosis and at risk of developing the disease [1]. Although there is dramatic advancement in diagnostics and therapeutics in the field of medicine, tuberculosis continues to affect mankind while millions of people suffering and dying from tuberculosis. In Sri Lanka, tuberculosis still remains a major public health problem. Although cases of pulmonary tuberculosis are declining owning to the advancement in diagnostics and improvement in public awareness, the incidence of extrapulmonary tuberculosis remains a diagnostic enigma due to protean of atypical presentation.

According to the WHO definition, extrapulmonary TB is defined as tuberculosis affecting outside the pulmonary parenchyma [2]. It presents 20–25% of cases of tuberculosis [3]. The common sites are lymph nodes, osteoarticular, gastrointestinal, central nervous system, and genitourinary tract [4]. Tuberculosis can involve any part of the gastrointestinal tract, and it comprises 3% of all cases of extrapulmonary tuberculosis [5]. Ileocecal involvement is the commonest. Ramirez et al. described several mechanisms of intestinal tuberculosis which includes consumption of contaminated milk/food by *Mycobacterium bovis,* swallowing of infected sputum in a patient with pulmonary tuberculosis, hematogenous spread from a case of active tuberculosis patient, or locoregional spread from an adjacent focus [6]. Presentation of intestinal tuberculosis is very nonspecific leading to delayed clinical diagnosis. Our patient did not have a past history of tuberculosis elsewhere. He denied longstanding cough or hemoptysis, and his chest X-ray was unremarkable.

Appendicular involvement in gastrointestinal tuberculosis is a rarity. It is reported more commonly in association with the ileocecal tuberculosis, but in the absence of any evidence of tuberculosis elsewhere by extensive imaging and at laparotomy, isolated appendicular involvement could be considered as primary appendicular tuberculosis [7, 8]. We believe that our case could represent the latter form of isolated appendicular involvement as the imaging of the gut with a CECT, and close examination of the bowels at the laparotomy did not reveal any evidence of ileocecal or any other involvement of the gut.

Three types of appendicular involvement in tuberculosis are described in the literature [9]. The first type is acute inflammatory type similar to pyogenic appendicitis, due to rapidity and severity of the clinical presentation, this group undergoes surgery early, macroscopy is indistinguishable from acute bacterial form, and diagnosis is made at histologist's bench. Second form is a subacute-to-chronic form presenting with vague abdominal complaints such as pain, diarrhea, or inflammatory mass. Third form is incidentally diagnosed at the histology of appendicectomy specimens during unrelated surgeries. Our patient had subacute illness with fever and diarrhea with minimal abdominal pain, laparotomy was done based on CT findings, and in the laparotomy, the appendix was minimally inflammed, so he belongs to the subacute form of appendicular tuberculosis.

Diagnosis of appendicular tuberculosis is difficult owning to the nonspecific clinical presentation and lack of definite noninvasive tests. At the presentation of the patient, intestinal tuberculosis was considered in the differentials, but most of the noninvasive tests were not in favor of TB. His Mantoux test was negative, and there were no features of active or past TB in the chest radiograph. Interestingly, he had moderate amount of free fluid in the abdomen. The unexplained lymphocytic ascites with an SAAG  <1.1 g/dl is classically described in tuberculous ascites [8]. However, in our patient, ascites was a lymphocytic transudative ascites with an SAAG of 1.5 g/dL. The TB PCR assay in peritoneal fluid was negative for tuberculosis. No other cause for a transudative ascites was evident in the extensive diagnostic workup which excluded liver cirrhosis, portal hypertension, or portal vein thrombosis. Transudative ascites in TB peritonitis is extremely rare. In the literature, it is reported in few case reports. Wariyapperuma et al. described a Sri Lankan lady with biopsy and microbiologically proven TB peritonitis with a transudative ascites [10]. She had concomitant portal vein thrombosis which could have partially contributed for transudative ascites. Another very interesting work by Manohar et al. studied 145 patients with TB peritonitis, and the peritoneal fluid analysis revealed an exudate (total protein: 30 g/l) in 96–4% and a transudate in 3–6% of patients, with a mean protein content of 48 g/l [11]. Although the reason for having a transudative ascites in an inflammatory condition such as gut tuberculosis is not explained explicitly in the literature, we believe that persistent low-grade inflammation and localized nature of appendicular tuberculosis could have contributed for the low protein content in the ascites. We emphasize that transudative nature of the ascites should not be used as an exclusion criterion for tuberculosis of the gut.

In the diagnosis of the tuberculous appendicitis, radiology may have a limited role. Ultrasonography and CT might pick an inflamed appendix, peritoneal or mesentery involvement, and presence of free fluid, but specific clues for an etiological diagnosis for TB may not be possible with imaging alone. Histology plays the main role in the diagnostic workup. As in this case until histology confirmed TB of the appendix, it was consider lower in the differentials due to poor support from other hematological, biochemical, and imaging investigations. Histological features of the TB appendix are similar to the rest of the gut which involves presence of tuberculous granulomata in the mucosa, submucosa, and muscle layers of the appendix consisting of epithelioid cells, Langerhans type, lymphocytes, mononuclear cells, and central area of caseous necrosis [12].

Treatment of appendicular tuberculosis is similar to other extrapulmonary tuberculosis which involves the standard treatment of isoniazid, ethambutol, rifampicin, and pyrazinamide for a duration of 6–9 months. In the initial part of the period, we used dexamethasone as an adjunct to reduce the complications of abdominal TB such as adhesion formation and fibrosis. There is mixed evidence on use of corticosteroids in the management of abdominal TB. A study by Alrajhi provides strong evidence for reduction of complications of abdominal TB following use of steroids [13]. However, this study is neither randomized nor blinded. A review by Haas et al. suggests a modest benefit of steroids in reducing late intestinal obstruction but emphasizes the need of further randomized prospective studies [14]. Recent meta-analysis by Soni et al. on use of steroid in intestinal tuberculosis concluded that the available date is limited to peritoneal tuberculosis and questioned the generalisability of the results owning to the poor quality of the studies [15]. As there was no evidence of intestinal obstruction clinically and in the CT scan of the abdomen and normal macroscopic findings of the ileocecal region, we believe that anti-tuberculous treatment alone would be enough and further surgical resection would be unnecessary.

## 4. Conclusion

Tuberculosis involving the appendix is a very rare form of extrapulmonary tuberculosis which poses a diagnostic dilemma in treating physicians owning to its protean manifestations. High degree of suspicion and prompt histological assessment is the key to diagnosis. Presentation may be very atypical, and initial tests may not prompt the diagnosis of tuberculosis, but histological assessment will guide the physician to the correct diagnosis and the prompt treatment would confer excellent prognosis. We would strongly recommend the need of histological assessment of all routine appendicectomy specimens.

## Data Availability

No data were used to support this study.

## Abbreviations

TB: Tuberculosis
PCR: Polymerase chain reaction
ALT: Alanine transaminase
AST: Aspartate transaminase
LDH: Lactase dehydrogenase
CRP: C-reactive protein
SAAG: Serum-ascites albumin gradient
CECT: Contrast-enhanced computed tomography
ATT: Anti-tuberculous therapy.
